# Compulsory Segregation of Consumptives

**Published:** 1919-08-09

**Authors:** 


### COMPULSORY SEGREGATION OF CONSUMPTIVES.

### Powers of Health Authorities.

Southwark Borough Council, which has long been anxious to secure the segregation of persons in an advanced stage of consumption, has now had a comprehensive report prepared by the Town Clerk, the Medical Officer of Health, and the Tuberculosis Officer, on the following special points :—

(1) The question of the provision by the State of accommodation other than that provided by institutions maintained by voluntary charitable bodies, in which persons in an advanced stage of consumption can end their days; and

(2) The question of compulsory powers being given to local authorities to remove cases where it can be shown that the patients, having regard to their surroundings, are a danger to the health of those with whom they reside.

The report recalls, with reference to the first point, that as far back as 1913, at a conference of London sanitary authorities convened by the London County Council, attention was drawn to the necessity of providing "rest-homes," and it was pointed out how undesirable it was that advanced consumptives should be mixed with more hopeful cases. The question was also considered in 1918 by another conference, when the opinion was expressed that greater attention should be paid to the treatment of advanced and incurable cases for which inadequate provision is made.

The report proceeds to explain that the only State provision at present is accommodation in a Poor-Law institution, and a number of beds for discharged soldiers provided by arrangement with the Insurance Commissioners.

Very few consumptives will voluntarily enter a workhouse infirmary. The accommodation provided by voluntary institutions for such cases is quite inadequate for the need. It is generally admitted that segregation and housing of advanced cases should be undertaken by local authorities and accommodation provided free from the pauper taint so much dreaded by the unfortunate patients.

With regard to the second point, compulsory power of removal by local authorities in respect of certain cases of infectious disease already exist under the Public Health (London) Act, 1891, but this does not extend to tuberculosis. Article 7 of the Tuberculosis (in Hospital) Regulations, 1911, provides that nothing in the Regulations shall authorise or require a medical officer of health or a local authority to put in force any enactment which renders a consumptive or the person in charge of the patient or any other persons liable to a penalty, or subjects the patient to any restriction, prohibition, or disability affecting himself or his employment, occupation, or means of livelihood on the ground of his suffering from tuberculosis.

Compulsory powers of removal have, however, been obtained by some of the provincial Councils under private Acts of Parliament; for example, the St. Helen's Corporation Act of 1908.

The Public Health (Prevention and Treatment of Disease) Act, 1913, also contains a special clause dealing with treatment of tuberculosis. Section 3 empowers any sanitary authority to make any such arrangements as may be sanctioned by the Local Government Board for the treatment of tuberculosis; this power to be in addition to and not in derogation of any other power. If this section be acted upon it may happen that one borough would go in for compulsory removal of advanced cases, whilst another would not act so drastically. Having regard to the fact that a certain percentage of consumptive cases are a danger and cause infection to others, and refuse to enter an institution or take steps to prevent the spread of the disease, compulsory removal should be general throughout the country in proper cases, and the Tuberculosis Regulations of 1911 should be so extended that a medical officer of health should have power to order with due legal precautions the compulsory removal of a case where it is clearly proved that such a case is tending to spread the disease, and the affected person refuses to take advantage of voluntary measures offered.

